# Perinatal immuno/inflammatory responses in the presence or absence of bovine fetal infection

**DOI:** 10.1186/s12917-018-1652-4

**Published:** 2018-11-01

**Authors:** Paulina Jawor, John F. Mee, Tadeusz Stefaniak

**Affiliations:** 1 Department of Immunology, Pathophysiology and Veterinary Preventive Medicine, Wroclaw University of Environmental and Life Sciences, 31 C.K. Norwida, 50-375 Wrocław, Poland; 2 Animal and Bioscience Research Department, Teagasc, Moorepark Research Centre, Fermoy, Co. Cork, Ireland

**Keywords:** Acute phase proteins, Immunoglobulins, Interleukin-6, Stillborn, Infection in utero

## Abstract

**Background:**

It is known that the bovine fetus can mount an immune and inflammatory reaction to infection, but it is not known whether there is a contemporaneous maternal response. Nor is it known whether the response of calves which die perinatally, with or without infection, differs from that of live perinates. Hence, the objective of this study was to determine if acute phase reactant and immunoglobulin concentrations differed between calves (and their dams) in three groups: live calves (CC; *n* = 21) and dead calves with (PM INF+; *n* = 22) or without (PM INF-; *n* = 89) in utero infection. In calf plasma, serum amyloid A, haptoglobin, immunoglobulins M, G_1_ and G_2_ and interleukin-6 were measured. In dam serum, SAA and Hp was measured and in amniotic and abomasal fluid, IL-6 was measured.

**Results:**

Live calves had higher plasma concentrations of SAA and IL-6 than dead calves with (PM INF+) or without (PM INF-) in utero infection. Calves in the PM INF-, but not PM INF+ group, had higher Hp concentrations than calves in the CC group. Calves in the PM INF+ group had higher IgG_1_ concentrations than calves in the PM INF- and CC groups. Except for higher IgG_1_ and IgG_2_ concentrations, biomarker values did not differ significantly between dead calves with or without in utero infection. Live calves had higher IL-6 concentrations in abomasal fluid compared to PM INF- calves. There were no significant differences in blood biomarker concentrations between dams of the three groups of calves. Amniotic fluid IL-6 concentrations were higher from the dams of control calves than the dams of uninfected calves.

**Conclusions:**

Differences in biomarkers (higher Hp and IgG_1_; lower SAA and IL-6) between perinatal mortalities and live perinates probably reflect differences between these two groups in age at sampling (SAA and IL-6) and in utero infection (IgG_1_). Out of the six analytes measured in calves, only IgG_1_ and IgG_2_ were biomarkers of (chronic) in utero infection.

## Background

Bovine perinatal mortality (PM) may be defined as the death of a calf/calves prior to, during, or up to 48 h after birth, following full-term pregnancy (≥260 d) [1, 2]. Perinatal mortality rates internationally vary between 4 and 7% [3] and increasing stillbirth rates have been reported internationally [1, 4, 5]. While much of this loss has been attributed to dystocia [6], an increasing proportion of calf loss occurs at eutocia (‘the unexplained stillbirth’). Some of this loss is caused by infections [7]. But, given the limitations in detecting infectious agents in calf carcasses, current estimates of the proportion of loss attributable to infections may underestimate the extent of their contribution. However, instead of attempting to detect infectious agents, it may be possible to use biomarkers of infection to explain some of this currently unexplained mortality.

The acute phase response is a non-specific, multifactorial response which develops after any disturbance of homeostasis, in particular during inflammation, designed to remove and replace damaged tissue. Damage to tissue activates cells which release acute phase cytokines including, in decreasing order of importance; interleukin-6 (IL-6), interleukin-1 beta (IL-1b) and tumor necrosis factor-alpha (TNF-a) [8]. The systemic reaction to proinflammatory cytokines is production and secretion of acute phase proteins (APPs) by the liver [9]. In cattle immunoglobulins (Igs) from the dam do not cross the placenta and high fetal serum immunoglobulin (Ig) concentrations are strong indicators of fetal infection [10].

In human medicine the reaction of fetus to inflammatory stimuli is described as the fetal inflammatory response syndrome (FIRS) and is diagnosed by elevated IL-6 concentration in fetal blood. FIRS is typically diagnosed in pre-term fetuses, but can also occur in term fetuses [11, 12]. Because APPs are released early in the inflammatory response, testing for APPs allows early detection of inflammation. Hence, APPs have been widely used as biomarkers for detecting sick animals [13], monitoring of calf group health [14, 15], monitoring of response to treatment [16, 17], or to monitor the response to repeated liver biopsy [18, 19]. The most common APPs measured in cattle are haptoglobin (Hp) and serum amyloid A (SAA) [20].

Our recent research, showed, for the first time, that measurement of SAA and IgM concentrations may be used in the diagnosis of bacterial, but not of viral or parasitical infections in cases of bovine PM [21]. However, to date the APP and Igs concentrations in contemporaneous cases of PM, control live calves and their dams have not been examined. Thus, the hypotheses tested in cases of PM, control live calves and their dams in this study were (1) cases of PM without diagnosed infection in utero would exhibit a similar acute phase and immune response to control live calves, (2) cases of PM infected in utero would exhibit a greater acute phase and immune response than uninfected cases of PM or live control calves and (3) that the dams of these calf groups would not differ in acute phase response after calving. Hence, the objective of this study was to determine if APP and Ig concentrations differed between live calves and dead calves (and their respective dams) with or without diagnosed infection in utero.

## Results

The plasma concentrations of SAA, Hp, Igs M, G_1_, G_2_ and IL-6 are shown in Table 1. Live calves (CC), had higher plasma concentrations of SAA and IL-6 than dead calves with (PM INF+, SAA *P* < 0.05; IL-6 *P* < 0.01) or without in utero infection (PM INF-, SAA and IL-6 *P* < 0.01). Calves in the PM INF-, but not PM INF+, group had higher Hp concentrations than calves in the CC group (*P* < 0.05). Calves in the PM INF+ group had higher IgG_1_ median concentrations than calves in the PM INF- (*P* < 0.05) and CC (*P* < 0.01) groups and concentrations of IgG_1_ tended to be higher in PM INF- compared to CC group (*P* < 0.10). Immunoglobulin M and IgG_2_ values did not differ between control and dead calf groups. Except for higher IgG_1_ and IgG_2_ concentrations, biomarker values did not differ significantly between dead calves with or without in utero infection.

**Table 1:** Biomarkers in plasma of control calves and dead calves with and without in utero infection

Analyte	CC	PM INF+	PM INF-
	*n* = 21	*n* = 22	*n* = 89
SAA (mg/L)	19.3^Aa^ (24.7 ± 15.9)	13.8^b^ (15.9 ± 17.3)	6.3^B^ (9.4 ± 9.9)
Hp (mg/L)	0.0^a^ (1.2 ± 3.6)	0.2 (3.6 ± 5.0)	0.0^b^ (6.0 ± 13.1)
IgM (mg/L)	21.4 (26.8 ± 25.6)	29.2 (42.2 ± 47.8)	16.6 (61.1 ± 153.5)
IgG_1_ (mg/L)	4.2^A^† (9.2 ± 17.8)	45.1^Ba^ (56.5 ± 79.1)	9.5^b^† (59.3 ± 160.3)
IgG_2_ (mg/L)	4.1 (4.8 ± 5.1)	10.8^a^ (31.1 ± 40.2)	2.8^b^ (8.6 ± 20.9)
IL-6 (pg/mL)	307.0^A^ (304.1 ± 17.2)	200.9^B^ (229.2 ± 70.4)	200.8^B^ (229.8 ± 69.0)

Median (mean ± SD) concentrations; SAA serum amyloid A; Hp haptoglobin; IgG_1_ immunoglobulin G_1;_ IgM immunoglobulin M; IgG_2_ immunoglobulin G_2_; IL-6 interleukin-6; CC control calves; PM INF+ dead calves with infection in utero; PM INF- dead calves without in utero infection

^A, B^Values within a row with different uppercase superscripts differ (*P* < 0.01)

^a, b^Values within a row with different lowercase superscripts differ (*P* < 0.05)

^†^*P* < 0.1 and > 0.05

Significantly more calves in the CC group had high concentrations of SAA compared to the two PM calf groups (Table 2). The proportion of calves with high analyte concentrations for other biomarkers was not different between groups.

**Table 2:** Percentage of calves within groups above reference thresholds for SAA, Hp, IgM and IgG values

Analyte	Threshold	CC	PM INF+	PM INF-
	(mg/L)	*n* = 21	*n* = 22	*n* = 89
SAA*	≥29.5	38.1	18.2	4.4
Hp	≥100	0	0	1.1
IgM	≥52.1	4.8	22.7	20.2
IgG	≥160	0	18.2	1.1

Reference thresholds for serum amyloid a and IgM from Jawor et al. [21], haptoglobin from Richter [22] and IgG from Sawyer [23]. SAA serum amyloid A; Hp haptoglobin; IgG sum of IgG_1_ and IgG_2_ classes; CC control calves; PM INF+ dead calves with infection in utero; PM INF- dead calves without in utero infection

*P ≤ 0.0001 Pearson’s chi-square

Live calves had higher IL-6 concentrations in abomasal fluid compared to PM INF- calves (mean ± SD 319.6 ± 41.4 pg/mL vs. 238.9 ± 7.6 pg/mL; *P* < 0.05) and tended to have higher concentrations than PM INF+ calves (mean ± SD 232.5 ± 92.5 pg/mL; *P* < 0.10).

There were no significant differences in blood biomarker concentrations between dams of the three groups of calves (Table 3). However, amniotic fluid IL-6 concentrations were higher from the dams of control calves than the dams of uninfected calves (Table 3). None of the amniotic fluid samples came from dams of PM INF+ calves.

**Table 3:** Plasma SAA, Hp and amniotic fluid IL-6 concentrations from dams of control and dead calves

Analyte	D CC *n* = 21	D INF+ *n* = 21	D INF- *n* = 81
SAA (mg/L)	58.6 (124.1 ± 129.1)	91.4 (98.2 ± 37.1)	89.9 (120.1 ± 105.1)
Hp (mg/L)	20.2 (362.5 ± 575.2)	45.8 (185.3 ± 288.1)	37.2 (236.6 ± 414.3)
IL-6 (pg/mL)	340.6^a^ (331.1 ± 29.1)	NS	279.2^b^ (274.6 ± 55.3)

*NS* not sampled; median (mean ± SD) concentrations; *SAA* serum amyloid A; *Hp* haptoglobin; *IL-6* interleukin-6; D CC dams of control calves; D INF+ dams of dead calves with in utero infection; D INF- dams of dead calves without in utero infection

^a,b^Values within a row with different lowercase superscripts differ (*P* < 0.05)

## Discussion

The results generated here present a unique opportunity to contemporaneously compare the acute phase and immune response in calves which survive with those who die at birth, whether infected or not, and acute phase proteins in their dams. Previous work only examined APPs in dead or live calves [24] or APPs and Igs in perinatal mortalities [21], but not both together and not in their dams.

The most rapidly responding markers of the acute phase reaction (IL-6, SAA) were significantly lower in the dead (irrespective of infection status) compared to the live calves, while the slower responding marker (Hp) was significantly higher only in the dead calves without infection in utero compared to the live calves. Among Igs, IgG_1_ and IgG_2_ concentrations were highest in infected dead calves while IgM concentrations did not differ between groups. Apart from amniotic IL-6, biomarker values did not differ between dams of live and dead calves.

Many of these are surprising findings which in some cases support, but in others, refute our a priori hypotheses that (1) calves which died perinatally without diagnosed infection in utero would exhibit a similar acute phase and immune response to calves which survived, (2) PM calves infected in utero would exhibit a greater acute phase and immune response than uninfected calves with perinatal mortality or control calves which survived and (3) that the dams of these calf groups would not differ in acute phase response after calving. The results of this study refute hypothesis one (for most biomarkers, except IgM and IgG_2_) and two (for SAA, Hp, IgM, IL-6 and partly IgG_2_), but support hypothesis three.

Regarding hypothesis one, the main reason why live and uninfected dead calves did not have similar SAA values may lie in their ages; the live calves were older (up to 9 h old) than the dead calves (99% < 1 h) when blood samples were collected. Immediately after birth calf SAA concentrations are low [24, 25], but rise rapidly, peaking at day 7 and decreasing after approximately 3 weeks [24]. However, in the present study the perinatal mortalities did not have the time to mount a postnatal SAA response, similar to that found in the live calves. They also did not mount a prenatal APP response whether they were infected in utero or not. This may be explained in the infected fetuses by the chronic nature of the infections detected here. Infected fetuses had time (days to weeks) to mount a pathogen-specific antibody response, thus the rise in SAA would have occurred sometime before birth and had subsided back to normal concentrations at birth. In a previous study on perinatal mortalities [21], SAA concentrations in calves without infection were also low, and all of these calves also died within 1 h of calving. The absence of infection [21, 26] or colostrum feeding [24] in the live calves in the present study, rule out the two other most likely causes of higher SAA concentrations in the live calves. This is the first study to report SAA concentrations in live healthy newborn calves which had not consumed colostrum up to 9 h after calving thus establishing a new reference range for such newborn calves. Samples in previous studies were either taken within a very short time after calving (10 min) [25] or after colostrum feeding [24].

Contrary to hypothesis one (that calves which died perinatally without diagnosed infection in utero would exhibit a similar acute phase response to calves which survived), Hp concentrations were higher in uninfected dead calves than in live calves. Haptoglobin is a slowly reacting APP [27]. Thus it might be expected that concentrations would be similar or higher in live compared to dead perinates as they had more time to mount a postnatal Hp response. However, the opposite was the case; Hp concentrations were higher in the dead perinates. This suggests that some prenatal factors may have influenced this response. But, Hp concentrations were not significantly higher in infected compared to uninfected dead calves. This suggests the antigenic stimulation of in utero infection was insufficient to maintain prolonged elevated Hp concentrations. Irrespective of calf group, Hp concentrations were very low suggesting that differences or lack of differences in concentrations between groups may have limited biological significance.

In support of hypothesis one, apart from IgG_1_, Ig concentrations (M and G_2_) did not differ between live and uninfected dead calves. The presence of (low) Ig concentrations in all three groups’ pre-colostral samples is not surprising [23]. The bovine fetus has the ability to produce IgM and IgG after 90 and 111 days of pregnancy, respectively [28], and the older the fetus, the higher the concentrations of IgM and IgG [23]. Though numerically higher IgG_1_ concentrations were found in uninfected dead calves compared to live calves, these concentrations were very low suggesting limited biological significance.

Contrary to hypothesis one (that calves which died perinatally without diagnosed infection in utero would exhibit a similar acute phase response to calves which survived), IL-6 concentrations (plasma and abomasal fluid) were higher in live compared to dead calves. While it is known that concentrations of IL-6 peak in cows before calving [29, 30] and that bidirectional transfer of IL-6 occurs between the human fetus and mother [31], neither adequately explain the discrepancy in IL-6 concentrations between the live and dead calves reported here. And while lower concentrations of IL-6 are secreted by human trophoblast cells under hypoxic compared to normoxic conditions [32], paradoxically, studies in human infants show higher plasma IL-6 concentrations in cases of severe hypoxia at birth [33, 34]. Thus, while one may speculate that the perinatal mortalities in the present study suffered more severe hypoxia (and ultimately, anoxia) than the live calves, the effects of hypoxia on IL-6 concentrations in bovine perinates have yet to be elucidated. Given this lack of basic information on bovine perinatal IL-6 profiles it is suggested that the higher concentrations detected here may again reflect the time lag required for perinates to mount a significant IL-6 response after birth. Interleukin-6 is the most potent inducer of APPs and it alone is able induce significant production of SAA, but not Hp, in bovine hepatocytes [35]. Thus, higher concentrations of IL-6 in control calves stimulated higher production of SAA in control calves.

In hypothesis two it was posited that if PM calves were infected in utero they would exhibit a greater acute phase and immune response than uninfected live or dead calves; this was not generally supported by the data generated here. The main reason for this may lie in the type of infections detected in the calves. The infections detected were chronic in nature rather than acute (fetuses had time to mount a pathogen-specific antibody response) and none of the live calves was infected. A previous study in dead perinates only has shown that differences in the APP response are detectable between dead calves with acute, but not chronic, infections [21]. Thus it is perhaps not surprising that SAA concentrations were not higher in infected dead calves as both the chronic nature of the in utero infections and the older age of the live calves at sampling contributed to higher SAA concentrations in the live rather than the dead calves, irrespective of infection status.

Infected (dead) calves had numerically higher and uninfected dead calves had significantly higher Hp concentrations than live (all uninfected) calves. Given the chronic nature of the infections and the slow rising Hp values (concentrations remain above baseline levels for 10–15 days) [36], the higher Hp concentrations in infected dead calves support hypothesis two (that PM calves infected in utero would exhibit a greater acute phase response than uninfected live or dead calves). However, the significantly higher Hp values in the uninfected group refute this hypothesis. The large within-group variability in Hp values (as discernable by the median and mean values) may indicate that biological differences may not have been detectable at these very low Hp concentrations.

In support of hypothesis two (that PM calves infected in utero would exhibit a greater immune response than uninfected live or dead calves), infected (dead) calves had significantly higher concentrations of IgG_1_ and IgG_2_ than uninfected (dead) calves and significantly higher concentrations of IgG_1_ than live (uninfected) calves. These findings are in agreement with a previous report on infected and uninfected dead perinates [21]. High serum Ig concentrations, particularly IgG_1_, the most abundant Ig, are a good marker of fetal infection [10]. In calves there is a lag phase between fetal infection and detectable Ig concentrations. For example, in experimental studies, IgM is detectable approximately 2 weeks and IgG_1_ approximately 3 weeks after fetal infection with BVDv and as the interval between infection and cesarotomy increased, the concentration of IgG_1_ increased more compared to IgM in the majority of fetuses [37]. Consistent with these findings, in the present study, the lag time between infection and sampling influenced the IgG_1_ and IgG_2_ classes more than IgM.

Perinatal mortality calves with diagnosed infection in utero were posited in hypothesis two to have higher concentration of IL-6, as human fetuses with diagnosed infection in utero showed elevated IL-6 concentrations [12]. However, the opposite was the case here; live calves had higher concentrations. As IL-6 values did not differ between infected and uninfected dead calves, it is suggested that the higher values in the live calves reflected older age at sampling and the lack of difference between dead calf groups was due to the chronic nature of the infections. While increased IL-6 production is a part of the acute phase reaction, the half-time of IL-6 elimination in humans lasts from minutes to hours [38].

The results generated here supported our third hypothesis that the dams of live and infected and uninfected dead calves would not differ in acute phase response after calving. While high concentrations of APPs (SAA, Hp) were detected in the three dam groups, these were expected in cows immediately after calving [39, 40]. These results are not surprising if it is accepted that the factors affecting perinate APP concentrations (e.g. age at sampling, infection, calving trauma, colostrum feeding) are not collinear with those affecting maternal APP concentrations. The only analyte which showed similar trends in dams and calves was IL-6. Differences in IL-6 concentrations between groups were consistent for amniotic fluid, abomasal and plasma samples which may be due to bidirectional transfer of IL-6 between the dam and her fetus. Apart from one study, in which amniotic fluid IL-6 concentration from a calving with a *Salmonella Stanley-*infected dead calf [41] was 6-times higher than in live calves in the present study, there are no other bovine perinatal IL-6 studies. Thus the data generated here represent baseline values for IL-6 concentrations in newborn calves and their dams.

## Conclusions

Significant differences in biomarkers (higher Hp and IgG_1_; lower SAA and IL-6) between perinatal mortalities and live perinates probably reflect differences between these two groups in age at sampling (SAA and IL-6) and in utero infection (IgG_1_). Adjustment for age at sampling should be considered when interpreting acute phase protein and IL-6 concentrations in newborn calves. Additionally, the interval between infection and sampling affected some Ig classes more than others. Out of the six analytes measured in calves, only IgG_1_ and IgG_2_ were biomarkers of (chronic) in utero infection. While significantly higher IL-6 concentrations were found in samples from dams with live calves, the biological significance of these findings is unclear given the paucity of current knowledge on bovine perinatal IL-6 profiles.

## Methods

### Ethical authorisation

The study design was approved by the II Local Ethics Committee in the Wroclaw University of Environmental and Life Sciences (permission no. 23/2012, 58/2014, 60/2014).

### Animals

This prospective study was carried out on calves which died in the perinatal period (*n* = 121) and their dams (*n* = 110; 11 cows had twins) and live control calves (CC; *n* = 21) and their dams (*n* = 21). All animals in this study came from Polish commercial dairy farms. Material was collected over a 20-month period (11.2013–06.2015). Perinatal mortalities were defined as calves (66 females and 55 males) born following a gestation of ≥260 days which died before, during or within 6 h after birth without receiving colostrum. A case definition of death within 24 h was planned, but only two calves survived the first hour and no calves died between 6 and 24 h after birth. These calves were either Holstein-Friesian (*n* = 113) or Holstein-Friesian crossbreds (Simmental, Jersey, Limousin, Brown Swiss sires; *n* = 8).

Control calf inclusion criteria were the same gestation length as perinatal mortalities, singleton calving, Holstein-Friesian breed and assisted but not difficult calving. None of the control calf had received colostrum before sampling (6 females and 15 males). From this group six male calves (selected on the basis that the farmers agreed to sell the newborn male calf) were euthanized between 1.30 and 8.30 h after birth (without receiving colostrum). They were first premedicated with 1 mL (20 mg) xylazine (Sedazin®, Biowet Pulawy) and 1 mL (100 mg) ketamine (Bioketan®, Vetoquinol) IV and then they were euthanized with a mixture of pentobarbital and pentobarbital sodium, 160 mg/mL (Morbital®, Biovet Pulawy) IV at a dose of 48–96 mg/kg bw.

### Herds

The study was conducted in 30 herds located in the south-west of Poland. Herds were recruited on farms located within 2.5 h one-way driving distance from Wroclaw. Farm staff were provided with a mobile phone number to call the veterinary staff who collected the material as soon after calf death as possible, on all days and times (day and night). Details about farm management, herd records and the calving associated with the calf were collected at farm visits. The median herd size was 73 with a range of 1–1037 cows/herd and the mean (SD) inter-calving period and previous lactation milk yield was 415 (38) days and 8884 (1442) kg/cow/305 DIM, respectively. Fifteen herds were vaccinated against BVD alone (*n* = 7), BoHV-1 alone (*n* = 4) or against BVD and BoHV-1 (*n* = 4) in 2013–2015. Free-stall housing system was present on 14 farms, tie-stall housing system was present on 14 farms and both systems were present on two farms. Total mixed ration was fed on 18 farms and on 12 farms ingredients were fed separately.

PM cases and CC were collected from 29 and 5 herds, respectively. Between 1 and 35 cases of PM and between 2 and 7 CC were investigated per herd. The degree of calving assistance was recorded in seven categories (supplied to the farmers): unobserved, observed but not assisted, normal assisted calving without calving jack, normal assisted calving with calving jack, difficult calving with calving jack, difficult calving without calving jack (assistance by at least three people and/or a veterinarian) and caesarean section.

### Samples and laboratory examinations

All carcasses (perinatal mortalities and euthanized control calves) were subjected to systematic external and internal gross examinations according to the same project-specific protocol. Necropsies of all PM calves were performed 8 ± 3 h after calving. Necropsies of the six CC were performed within 6 ± 3 h after calving and within 2 h ± 40 min after death. Abomasocentesis was performed with a sterile needle and syringe to collect abomasal fluid immediately after opening the abdominal cavity. Blood was aseptically collected from PM and CC by syringe from a jugular vein and immediately dispensed into 5 mL lithium heparinized tubes (MEUS Srl®, Piove di Sacco, Italy, 18,648) and centrifuged (14 min; 1860 x g). Plasma samples were aliquoted and frozen at − 80 °C until analysed. Farm staff collected amniotic fluid from the amniotic sac at six calvings of PM calves and from 20 CC calvings. Blood was collected from the dams of PM calves when the carcass was collected. Blood from the dams of CC and their calves was collected within 9 h of calving. Maternal samples were collected from the coccygeal vessels into 9 mL tubes with lithium-heparin (Sarstedt, Germany, cat no. 02.1065.001) and 10 mL serum separator tubes (MEUS Srl®, Piove di Sacco, Italy, cat no. 18203). Maternal samples (blood and amniotic fluid) were centrifuged within 4 h of collection (14 min at 1860 g at room temperature). Plasma and amniotic fluid were aliquoted and frozen at − 80 °C until analysis.

In calf plasma, SAA, Hp, IgM, IgG_1_, IgG_2_ and IL-6 were measured. In dam serum, SAA and Hp was measured and in amniotic and abomasal fluid, IL-6 was measured.

SAA was analyzed by a multispecies ELISA (TP 802, Tridelta Development, Maynooth, Ireland) with intra- and inter-assay coefficients of variation (CV) of 17.4 and 6.1%, respectively, and a detection limit of 4.6 mg/L. Haptoglobin was determined by ELISA (#2410–7 Life Diagnostics Inc., Knypersley, UK) with intra- and inter-assay CVs of 5.9% and 7.8%, respectively. The detection limit was 7.8 mg/L. Interleukin - 6 was analysed by ELISA (Qayee-Bio) with intra- and inter-assay CVs of 4.9 and 3.5%, respectively. The detection limit was 124.4 pg/mL. Immunoglobulin M, G_1_ and G_2_ concentration was determined by ELISA (E10–101, E10–116, E10–117 Bethyl Laboratories, Montgomery, TX). The intra- and inter-assay CVs for IgM was 4.3 and 1.2%, for IgG_1_ 12.8 and 2.0% and for IgG_2_ 14.4% and 2.1%, respectively. The limits of detection for IgM, IgG_1_ and IgG_2_ were 6.9, 13.2 and 4.1 mg/L, respectively.

In calf plasma, pathogen-specific antibodies were measured: *Neospora caninum*, glycoprotein B of BoHV-1 and BVDv using the IDEXX Neospora Ab Test, IDEXX IBR gB X3 Ab Test and IDEXX BVDV Total Ab Test (Hoofddorp, The Netherlands), SBV using the Serological ID Screen Schmallenberg Virus Competition Multi-Species ELISA (ID-vet, Grabels, France), *Leptospira hardjo* and *Leptospira pomona* using live strains of *L. hardjo* and *L. pomona* in the microscopic agglutination test at the veterinary diagnostic laboratory as described in Jawor et al. [7] and *Coxiella burnetii* in the IDEXX Q Fever Ab Test (Hoofddorp, The Netherlands) (Krol et al., unpublished).

### Experimental groups

Calves and their dams were each divided into six mutually exclusive groups. Three calf groups were compared; two PM and one CC group. The first PM group (PM INF+; *n* = 22) included only calves in which pathogen-specific antibodies (BVDv, *n* = 3; *Coxiella burnetii, n* = 6; *Leptospira hardjo*, n = 3*, Leptospira hardjo* and *L. pomona*, *n* = 1; *Neospora caninum*, *n* = 5 or SBV, *n* = 4), but not antigens, were detected. Calves with infection in utero diagnosed by the detection of antigen/s [7] were excluded from the study as multiple infections and co-infections were detected and their number was too small for statistical analyses (*n* = 10). Diagnosis of infection was made only in the calves, not their dams. The second PM group (PM INF-; *n* = 89) included calves in which no infection (neither antibody nor antigen) was diagnosed. The third group was the control calves (CC; *n* = 21); infection in utero was not diagnosed in any of the CC. The majority of calvings in all three groups were assisted (CC 100%, INF+ 86% and INF- 92%). Three dam groups were compared; dams of PM INF+ calves (D INF+), dams of PM INF- calves (D INF-) and dams of control calves (D CC). Results from calves and dams were analysed separately.

### Statistical analysis

For each test, the experimental unit was an individual animal. Data were checked for normality using the Lilliefors test. All parameters had abnormal distributions therefore logarithmic transformation (log10) was performed. After transformation only SAA values in cows were normally distributed. SAA values were tested using ANOVA and post hoc comparisons were made using Tukey’s HSD test. Values without normal distributions were analyzed using the Kruskal-Wallis ANOVA with post hoc multiple comparisons of mean ranks in cases where three group were compared and the U-Mann-Whitney test was used in cases where two group were compared. The frequencies of cases above and below the normal threshold values for APPs and Igs were compared between groups by Pearson’s chi-square analysis. Statistical analyses were conducted using Statistica 12.5 (StatSoft Inc., Tulsa, OK, USA).

## Abbreviations

APP(s): acute phase protein(s)
CC: control calves
D CC: dams of control calves
D INF-: dams of dead calves without in utero infection
D INF+: dams of dead calves with in utero infection
FIRS: fetal inflammatory response syndrome
Hp: haptoglobin
Ig(s): immunoglobulin(s)
IL-6: interleukin 6
PM INF-: dead calves without in utero infection
PM INF+: dead calves with infection in utero*.*
PM: perinatal mortality
SAA: serum amyloid A
